# Supporting Local Public Health and Planning Professionals to Implement Built Environment Changes: A Technical Assistance Program to Promote Physical Activity in Texas

**DOI:** 10.5888/pcd21.230420

**Published:** 2024-06-20

**Authors:** Caroline Magee, Cari Browning, Ronald Stokes-Walters, Lauren Maxwell, Justin Buendia, Nimisha Bhakta

**Affiliations:** 1 Health Promotion and Chronic Disease Prevention Section, Texas Department of State Health Services, Austin

## Abstract

Built environment approaches that improve active transportation infrastructure and environmental design can increase physical activity. Funded by the Centers for Disease Control and Prevention, the Texas Department of State Health Services rejuvenated the Texas Plan4Health program from 2018 to 2023 to expand such approaches in Texas by providing technical assistance to teams of local public health professionals and planners to identify and implement projects connecting people to everyday destinations via active transport in their communities. However, the COVID-19 pandemic prompted Texas Plan4Health to modify the delivery of technical assistance to accommodate restrictions on travel and in-person gatherings. We used qualitative methods to conduct a postintervention process evaluation to describe the modified technical assistance process, understand the experiences of the 4 participating communities, and identify short-term outcomes and lessons learned. Texas Plan4Health helped communities overcome common barriers to built environment change, facilitated collaboration across community public health and planning professionals, and educated professionals about active transportation infrastructure and the relationship between their disciplines, thereby increasing community capacity to implement built environment improvements. This outcome, however, was mediated by the pre-existing resources and previous experiences with active transportation planning among the participating communities. Public health practitioners seeking to improve active transportation infrastructure and environmental design for physical activity should consider community-engaged approaches that advance partnership-building and collaborative experiential education among public health, planning, and other local government representatives, directing particular attention and additional training toward communities with fewer resources.

**Summary**

**What is already known on this topic?**

The Centers for Disease Control and Prevention recommends built environment approaches that increase physical activity by improving active transportation infrastructure and environmental design.

**What is added by this report?**

Technical assistance provided through a state-level intervention in Texas helped 4 participating communities develop multidisciplinary partnerships; increase knowledge of active transportation infrastructure and the relationship between public health and planning; and increase capacity to implement additional built environment improvements.

**What are the implications for public health practice?**

Community-engaged approaches to multidisciplinary partnership-building and experiential education can build community capacity for built environment improvements.

## Introduction

Built environment features such as walking and cycling infrastructure, connected streets, and mixed land use are associated with increased physical activity and reduced risk of chronic disease (1). In December 2016, the Community Preventive Services Task Force found sufficient evidence to recommend built environment approaches that increase physical activity by improving active transportation infrastructure and land use or environmental design (2). Accordingly, CDC’s Division of Nutrition, Physical Activity, and Obesity (DNPAO) revised their priority strategies to require State Physical Activity and Nutrition (SPAN) 2018–2023 grant recipients to implement policies and activities that increase physical activity by “connecting people to everyday destinations” (CPED) via active-friendly routes (3). As 1 such grant recipient, the Texas Department of State Health Services (DSHS) Obesity Prevention Program collaborated with public health and planning professionals through the American Planning Association Texas Chapter (APATX) to implement the Texas Plan4Health: Connecting People to Everyday Destinations intervention to support local Texas communities in developing and executing their own CPED projects.

## Purpose and Objectives

The purpose of our intervention was to help Texas communities implement their own built environment changes that support active transportation and CPED. The intervention originally intended to offer yearlong in-person training and technical assistance to Texas communities, provide interdisciplinary training to regional public health and planning professionals, and garner interest among statewide public health and planning leaders in CPED and the intersection between public health and planning. However, the COVID-19 pandemic prompted Texas Plan4Health leaders to restructure intervention activities and deliver training and technical assistance online. Given these changes, we conducted a postintervention evaluation to describe the modified training and technical assistance process, understand the experiences with the modified intervention among Texas communities, and identify short-term outcomes and lessons learned. The objective of this evaluation was to inform the advancement of a statewide CPED training and technical assistance program for Texas communities and guide public health practitioners seeking CPED improvements in their communities.

## Intervention Approach

DSHS recognized that Texas public health professionals and planners needed support, training, and tools to successfully implement CPED as a new SPAN priority strategy in 2018. APATX and the Texas Public Health Association stood out as key partners with the needed interdisciplinary expertise, statewide relationships, and background. With national CDC Plan4Health grant funding (4), leaders from APATX and the Texas Public Health Association had successfully collaborated in 2017 to implement their Plan4Health roundtable model in Van Zandt County, a rural Texas community recovering from tornadoes. The roundtable convened public health and planning professionals and applied their complementary skills to create a disaster planning and recovery toolkit for rural communities (5). When CDC Plan4Health grant funding ceased in 2018, DSHS contracted with APATX to expand the application of the Texas Plan4Health model from disaster preparedness to CPED with SPAN grant funding beginning in October 2019.

The Texas Plan4Health intervention had a 3-pronged approach. Public health and planning leaders would

1. Deliver in-person training and technical assistance based on the CDC Plan4Health model to help 8 communities develop multidisciplinary coalitions and CPED projects;
2. Train additional teams of planners and public health professionals to become regional Texas Plan4Health training and technical assistance facilitators; and
3. Offer professional development sessions at annual APATX and Texas Public Health Association state conferences.

However, in March 2020, the COVID-19 pandemic forced Texas Plan4Health leaders to pause community recruitment and delivery of training and technical assistance to their first community. To navigate pandemic conditions, from September 2020 to September 2021, Texas Plan4Health leaders participated in Walkability Virtual Academy training (6). From January 2021 to September 2021, they resumed training and technical assistance with their first community virtually and offered monthly group training and technical assistance calls to 18 local health departments working on physical activity action planning. In September 2021, Texas Plan4Health community recruitment resumed, and the 3-pronged intervention pivoted to the following:

1. Deliver hybrid training and technical assistance informed by Walkability Virtual Academy trainings and an affiliated DNPAO consultant to help 4 communities develop multidisciplinary coalitions and CPED projects;
2. Collaborate with 1 community to host a 2-day in-person regional conference to train additional teams of planners and public health professionals to develop and implement community CPED projects, considering regional connectivity; and
3. Offer professional development sessions at statewide conferences, in collaboration with the Walkability Virtual Academy–affiliated DNPAO consultant.

Throughout the project, pandemic economic inflation prohibitively increased operational expenses, leading Texas Plan4Health to cease 9 months early, in November 2022, due to inadequate funds.

## Evaluation Approach

The postintervention process evaluation used qualitative methods to describe the modified training and technical assistance process, evaluate its perceived usefulness to participating communities, and identify factors that supported or hindered intervention aims. The primary DSHS evaluator (C.M.) conducted semistructured interviews with the Texas Plan4Health leaders in January 2023 and semistructured focus groups with each of the 4 participating communities in May and June 2023. Interviews guided development of the focus group participant list and discussion guide and were used to validate intervention delivery timelines and steps identified in focus groups. Interviews included questions about CPED needs, key allies, and activities in each community, and what factors they thought facilitated or impeded their work. Focus group discussions centered on understanding each community’s experience of the intervention and included questions about the community’s CPED needs, their CPED project(s), what the Texas Plan4Health team did, what aspects of the training and technical assistance were useful or not useful, what they learned from participating, and whether they felt they could develop and lead a CPED project on their own. All interviews and focus group sessions were summarized by a notetaker (R.S-W.) and were recorded and transcribed after informed consent was obtained from participants. The primary evaluator (C.M.) used grounded theory (7) to code and analyze the focus group transcripts in Atlas.ti Windows version 23.2.3.27778 (ATLAS.ti Scientific Software Development GmbH). A second evaluator (R.S-W) independently coded the focus group transcripts in Atlas.ti using a codebook based on the first evaluator’s coding. The 2 evaluators discussed coding disagreements until they reached consensus. Institutional review board approval was not required, per DSHS policy.

## Results

Four communities participated in the Texas Plan4Health intervention (Table 1). Communities varied in their previous experience and resources for CPED efforts. More-resourced communities (Tyler, Paris) had pre-existing interdisciplinary relationships, support from local political leaders, and active transportation plans and projects, while less-resourced communities (Mathis, Eagle Pass) lacked these characteristics.

**Table 1:** Characteristics of Communities Participating in the Texas Plan4Health Training and Technical Assistance Intervention

Characteristic	Community			
	Tyler	Mathis	Paris	Eagle Pass
**Population^a^**	109,510	4,206	25,032	28,120
**Location**	Northeast Texas, 100 miles east of Dallas	Lower Gulf Coast, 35 miles northwest of Corpus Christi	Northeast Texas, 105 miles northeast of Dallas	Texas and Mexico border, 140 miles southwest of San Antonio
**Public health professionals**	1 Regional public health department professional who participated in the Texas Plan4Health roundtable focused on disaster preparedness in 2017	2 County public health department professionals with backgrounds in community health education	1 County health district professional	2 Regional public health department professionals (Eagle Pass has no city or county public health department)
**City planners**	1 City planning director and 1 Metropolitan Planning Organization planner	NA; Mathis has no city planner	1 City planning director and 1 city engineer	1 City planning director and 2 city engineers
**Other personnel**	NA	NA	City mayor, city historic main street coordinator	NA
**Pre-existing cross-disciplinary partnerships**	Yes	No	Yes	No
**Status of comprehensive plan at intervention start date**	Earlier in 2019, the Metropolitan Planning Organization had adopted a comprehensive active transportation plan providing data-driven recommendations to guide the development of pedestrian and bicycle infrastructure in the greater Tyler area	The comprehensive plan in Mathis had not been updated in >25 years	Comprehensive plan updates in progress at the time of the intervention	None reported
**Other active transport projects at intervention start date**	None reported	None reported	Numerous downtown street development projects in progress at the time of the intervention, supported by the Texas Historical Commission Main Street Program (8) and the NorthEast Texas Trail Coalition (9)	None reported
**Training and technical assistance delivery period and modifications received**	2019–2021. Received in-person training and technical assistance from October 2019–March 2020, then virtual training and technical assistance.	2020–2021. Received all virtual training and technical assistance. The monthly group TA calls that Texas Plan4Health leaders facilitated with local health departments replaced Steps 1 and 2 in the training and technical assistance process for Mathis.	2021–2022. Received mostly virtual training and technical assistance, with 1 in-person visit. The public health professional participated in the monthly group TA calls that Texas Plan4Health leaders facilitated with local health departments, supporting Step 1 of the training and technical assistance process.	2022. Received all virtual training and technical assistance. The public health professional participated in the monthly group TA calls that Texas Plan4Health leaders facilitated with local health departments, supporting Step 1 of the training and technical assistance process. Eagle Pass did not complete Step 5 of the training and technical assistance process because Texas Plan4Health ended earlier than expected.
**In-person Texas Plan4Health professional development sessions attended**	APATX Conference 2021 (walk audit workshop and plenary session “Planning for Healthy Communities” led by CDC DNPAO consultant).	APATX Conference 2021 (walk audit workshop and plenary session “Planning for Healthy Communities” led by CDC DNPAO consultant). Also gave panel presentation on virtual walk audit process and resulting CPED project.	APATX Conference 2021 (walk audit workshop and plenary session “Planning for Healthy Communities” led by CDC DNPAO consultant). APATX Conference 2022. Hosted and attended a 2-day in-person regional conference to train teams of planners and public health professionals to develop and implement community CPED projects, considering regional connectivity.	APATX Conference 2022

Abbreviations: APATX, American Planning Association Texas Chapter; CPED, connecting people to everyday destinations; DNPAO, Division of Nutrition, Physical Activity, and Obesity; NA, not applicable; TA, technical assistance.

a Population estimates for January 1, 2023, from the Texas Demographic Center (10).

Qualitative analysis of the focus group transcripts identified 5 main steps in the Texas Plan4Health training and technical assistance process (Table 2). In addition to these steps, Texas Plan4Health leaders gave presentations on CPED concepts; recommended grants, Plan4Health-facilitated conferences, and workgroup opportunities; and shared educational resources on CPED, including a walk audit toolkit and debriefing guide. Texas Plan4Health leaders also connected Mathis to a university initiative that pairs urban planning graduate students with communities that need planning services (11).

**Table 2:** The 5 Steps of Texas Plan4Health Training and Technical Assistance Project as Identified in Focus Groups and Supporting Quotes From Focus Group Participants

Step	Description	Supporting quotes from focus groups
**1. Identified multidisciplinary community allies**	Texas Plan4Health leaders met with community representatives and potential allies. Community representatives assessed and discussed current active transportation policies, plans, or projects (if applicable) and community priorities. Texas Plan4Health leaders provided guidance on how to engage allies and types of allies to engage, like community champions, other representatives from health and planning departments, and the TXDOT. Community representatives drafted a list of allies to invite to participate in steps 2 through 4.	We knew we probably would need to contact specific people but were not sure how do we start. This is not something that we’d ever done out of our department before. We do community health education and outreach. So, this was a totally different type of grant [Community representative 1, Mathis].
		We had an initial meeting to talk about the goals of the project and what we were trying to do. So, they helped us identify the different types of community stakeholders that we should be engaging with. So, we submitted a list saying, “Okay, well these are the ones that we think would be great for our community.” . . . We had the city of Eagle Pass that we were going to be working with, we had the TXDOT, we had also identified the school district, and then we had some, you know, community champions is what they wanted us to also look to see if we could — someone who would help us rally and get those partners involved [Community representative 1, Eagle Pass].
**2. Convened multidisciplinary community allies**	Texas Plan4Health leaders met with the community representatives and multidisciplinary allies to 1) facilitate a discussion of community priorities and brainstorm project ideas, 2) present the walk audit process (orally and/or with a toolkit), and 3) choose a walk audit location (or locations) based on community priorities and needs.	[Plan4Health] helped bring different community stakeholders together in the downtown area and talk to them about the project we were working on, and really were acting kind of as a catalyst to put the planners and the health care professionals together to work on this project and develop an outcome [Community representative 1, Tyler].
		We reached out to the different stakeholders to bring them on board, and Plan4Health helped us coordinate those meetings and facilitate the discussions for the project and helped identify [next steps] — so we did a walking audit, but they gave us the tools for that walking audit beforehand [Community representative 1, Eagle Pass].
		We also had a very productive facilitation on what our priorities were, what the needs were. And then we drilled down into . . . the main project with the team [Community representative 1, Paris].
**3. Conducted a walk audit**	Texas Plan4Health leaders, community representatives, and multidisciplinary allies walked through a predefined area of town, noting elements that support or inhibit different modes of active transportation for diverse community needs and the accessibility of everyday destinations by active transportation.	After we did do the first initial walk audit, that’s when we noticed there was a lot of areas that we could work on. [We] are both from this community, so we were aware of them, but we weren’t aware of the extent of what was needed. . . . So, the way we started, we did almost a Safe Routes to School type of walk audit of it. We started near the school area since they are close in proximity to each other. We started there and we noticed when kids would walk to school that there’s not really much of a sidewalk for them to walk on, there’s not much signage, there’s not much crosswalk markings on the ground or anything like that, and they kind of have to walk in the grass or walk in the road and stuff like that. So, it’s not really safe for them. And also, the speed limit’s also high in some of the areas, so that was also another thing. And then the areas that did have sidewalks, they were uneven, grass was growing through them, or there’s cracks and stuff like that. So, there was just a lot of needs [Community representative 2, Mathis].
		After we received a lot of the information, [the walk audit] gave us a new way to look at the connectability of different areas we had, what things make it more attractive, which things make it more safe. . . . How to connect people to their everyday destinations — that was the main focus of our course, our learning; and not just with a car, but even connecting to schools, children. We had a couple of areas where there were sidewalks to the street, but no really protective-looking crosswalk for the kids at the middle school. And those kind of things just were really pointed out, and all of us actually were more noticing that with the education that we received [Community representative 2, Paris].
**4. Facilitated a post-walk audit discussion and selected a project**	Texas Plan4Health leaders collaborated with the community representatives and multidisciplinary allies to 1) debrief the walk audit experience, 2) discuss different approaches to improving built environment or design elements that inhibit different modes of active transportation for diverse community needs or limit access to everyday destinations via active transportation, and 3) select an approach or approaches to improve CPED (ie, the proposed project).	I know we discussed what we experienced . . . the information that they had based off of what we experienced, and how we can pull that together and have a project [Community representative 2, Tyler].
		And I remember poring over a lot of maps with areas, markers, and a lot of studying on this. And there’s a lot of familiarization of neighborhoods. In Tyler specifically, I remember how they talked about the different designations that exist and how each one of them, you know, could be interconnected [Community representative 3, Tyler].
		There was a ‘lessons learned’ or ‘how would we take this to the next level’ conversation. And I think that was really helpful as well. You know, especially, if anything, maybe even giving us more ideas of how we could do it ourselves. But also, I’m sure it informed them as they went to other communities to work on similar projects [Community representative 1, Tyler].
**5. Supported community representatives to execute their chosen project**	Plan4Health leaders provided personalized, hands-on training and technical assistance to the community representatives and allies as they executed their proposed project. Training and technical assistance activities included 1) serving as extra expert staff directed by the community representatives and allies, 2) working alongside the community representatives as a coach that provides encouragement and expert professional guidance on CPED techniques and concrete steps to take to achieve project goals, and/or 3) providing specialized services that are beyond the community representatives’ skill set.	They were always our cheerleaders, and always telling us what we were doing good and what we could do to improve. . . . After we did the initial walk audit and we gathered the information and data from it, and I was like, “This is where we need to work. This is what our main focus should be for our project” and stuff like that. . . [T]hey were very supportive in the sense of like, “Yes, that sounds like a wonderful project. That sounds like a wonderful idea for your community. How can we help?” is always what [the Plan4Health leaders] always said to us in our meetings, like, “How can we help you? What do you need us to help you with?” [Community representative 2, Mathis].
		[The] person power, or manpower, of people to have a focus on some of our needs. . . [c]apacity and then also the attention and knowledge base on how to go about what we were wanting to achieve. So, might be pretty basic, but I mean it’s definitely helpful to have that technical expertise, to have the background to pull things together [Community representative 2, Tyler].
		[A Texas Plan4Health leader] also helped with the renderings. He was able to take Google street views or different aerials and overlay different types of bollards or planters or markings onto it. Those type of things are really good to show our city leadership, city council, or even the potential stakeholders, like in that case, the mall property owners, a visual of what we were trying to do. So that was really helpful. We’re not too technical, so . . . that’s something I don’t know that we could have done on our own [Community representative 2, Eagle Pass].

Abbreviations: CPED, connecting people to everyday destinations; TXDOT, Texas Department of Transportation.

### Participant reviews of the training and technical assistance process

All 4 communities emphasized the usefulness of the hands-on intervention approach, where professionals engaged in applied learning by identifying and executing a project from start to finish with guidance from Texas Plan4Health leaders. Across more- and less-resourced communities, participants valued the leaders’ technical expertise in walkability (Eagle Pass, Mathis, Paris), their accessibility (Mathis, Paris), and the extra staffing capacity dedicated to the project (Eagle Pass, Paris, Tyler). These components helped participants’ learning while addressing identified community barriers to accomplishing CPED projects —lack of time, personnel, or specialized professional skills. One participant said:

An approach where someone comes in, does some education, and then says, “Okay. Go.” That would be pretty hard for us to do with our capacity. But [Texas Plan4Health] was really knowing that you had that assistance to help walk you through and work side by side with you throughout the project [Community representative, Tyler].

Several useful intervention components were mentioned only by the 2 less-resourced communities. They included how affiliation with the Texas Plan4Health leaders and program gave their project credibility, facilitating buy-in from allies skeptical of their lack of resources (Mathis); and the program structure, which addressed the community team’s lack of time and coordination (Eagle Pass). The latter community said, “We wear a lot of hats here . . . we’re a lot of times pulled in different directions. . . . So, I really like these type of people with experience that have a really good system in place . . . [with] different checkpoints and milestones and keeping on track with the timeline” (Community representative, Eagle Pass).

Mathis and Eagle Pass expressed interest in participating in Texas Plan4Health again to feel more confident about making future CPED changes independently. The 2 more-resourced communities did not request this but agreed that longer-term community involvement with ongoing volunteer staff and additional funding would improve the intervention by better helping some participants sustain and expand their CPED projects.

### Intervention outcomes

Three communities reported the following educational outcomes: new technical knowledge about infrastructure, policies, and plans that support CPED and the implementation process (Eagle Pass, Mathis, Paris); new awareness of built environment characteristics and connectivity in their communities (Mathis, Paris, Tyler); and new recognition of the connection between planning, public health, economic development, and other disciplines (Eagle Pass, Paris, Tyler). Three communities also reported maintaining the new multidisciplinary partnerships they created during the intervention (Eagle Pass, Mathis, Tyler); and gaining a step-by-step process for developing and implementing CPED projects in the future (Eagle Pass, Mathis, Tyler). All but Eagle Pass, which did not complete the fifth step in training and technical assistance (ie, “supported community representatives to execute their project”), linked each of the identified short-term outcomes to a new belief in their capacity to implement CPED changes after the conclusion of the intervention. For example, 1 participant mentioned implementing a new CPED project postintervention and described how their new awareness of community trail linkages and technical knowledge about walkability infrastructure like “signage and delineators . . . helped empower us to know what to ask for and that would make a difference” (Community representative, Paris).

## Public Health Implications

The modified Texas Plan4Health training and technical assistance process informed by Walkability Virtual Academy training focused on multidisciplinary partnership-building and collaborative experiential learning about CPED. This process helped participating communities overcome barriers to implement CPED changes. It also yielded positive short-term outcomes that show potential for ongoing CPED changes. Communities that completed all 5 training and technical assistance steps attributed their perceived capacity to implement CPED changes to the knowledge, community awareness, interdisciplinary thinking, model for future projects, and partnerships gained from the intervention. The results illuminate the importance of strengthening multidisciplinary community partnerships and increasing baseline knowledge among planning and public health professionals about the relationship between their disciplines to build community capacity for built environment improvements (12). They also confirm that community-engaged intervention approaches can build community capacity for built environment improvements (13).

Our results highlight the difference in training and technical assistance needs between more-resourced communities and less-resourced communities. Tyler and Paris entered Texas Plan4Health with previously established CPED-relevant connections across government departments, support from local political leaders, and pre-existing active transportation plans and projects. These communities felt capable of sustaining postintervention CPED changes on their own. In contrast, Mathis and Eagle Pass lacked these resources and felt they needed ongoing support from Texas Plan4Health. This finding points to the importance of these resources in facilitating and sustaining community CPED changes, although completing (rather than not completing) the full training and technical assistance process and receiving hybrid (rather than virtual) training and technical assistance may also have influenced sustainability outcomes. The finding also indicates the need to bolster less-resourced communities with a longer intervention timeline or more intensive training and technical assistance focused on identifying allies and relationship-building, obtaining buy-in from local political leaders, and active transportation plans and policies.

The consistency of results and outcomes across community categories and training and technical assistance formats shows promise for the lower-cost scalability of a partially virtual intervention without sacrificing quality or efficacy. Conducting some training and technical assistance with groups of communities rather than individual communities, as Mathis and Paris experienced, may be another option for scaling or lengthening the intervention. However, training and technical assistance alone will not revolutionize the built environments of communities. All communities lack the necessary funding and staff to do this work; this lack of resources must be addressed to enable lasting change (14).

Public health and planning practitioners seeking to improve CPED should consider community engagement approaches to partnership-building and collaborative experiential learning for public health, planning, and other local government representatives, incorporating funding opportunities where possible. Practitioners should also consider offering regional or statewide opportunities to unite planning and public health professionals from multiple communities to participate in CPED professional development, discuss project ideas and roles, and experience in-person walk audit training. These approaches will spark local built environment improvements and build community capacity for future changes.
